# Supplementary material for: Spitzenkörper assembly mechanisms reveal conserved features of fungal and metazoan polarity scaffolds
Source: Nat Commun. 2020 Jun 5;11:2830. doi: 10.1038/s41467-020-16712-9 (PMC7275032; doi:10.1038/s41467-020-16712-9)
Supplement: Supplementary file 1 — Supplementary Information [file 41467_2020_16712_MOESM1_ESM.pdf]

Supplementary Information

Spitzenkörper assembly mechanisms reveal conserved features of fungal and metazoan polarity scaffolds

Zheng et al.

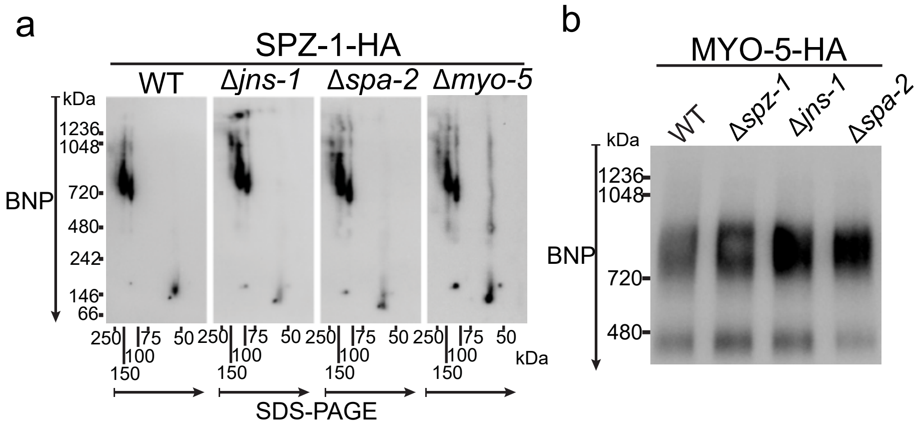

Supplementary Figure 1. SPZ-1 and MYO-5 migration in native-PAGE. a Extracts from the indicated strains were separated by blue native-PAGE (BNP), followed by second dimension SDS-PAGE. SPZ-1 is revealed by western against the HA-epitope tag. **b** Extracts from the indicated strains were separated by native-PAGE. MYO-5 is revealed by western against the HA-epitope tag. The direction of protein migration is indicated with arrows. Source data are provided as a Source Data file.

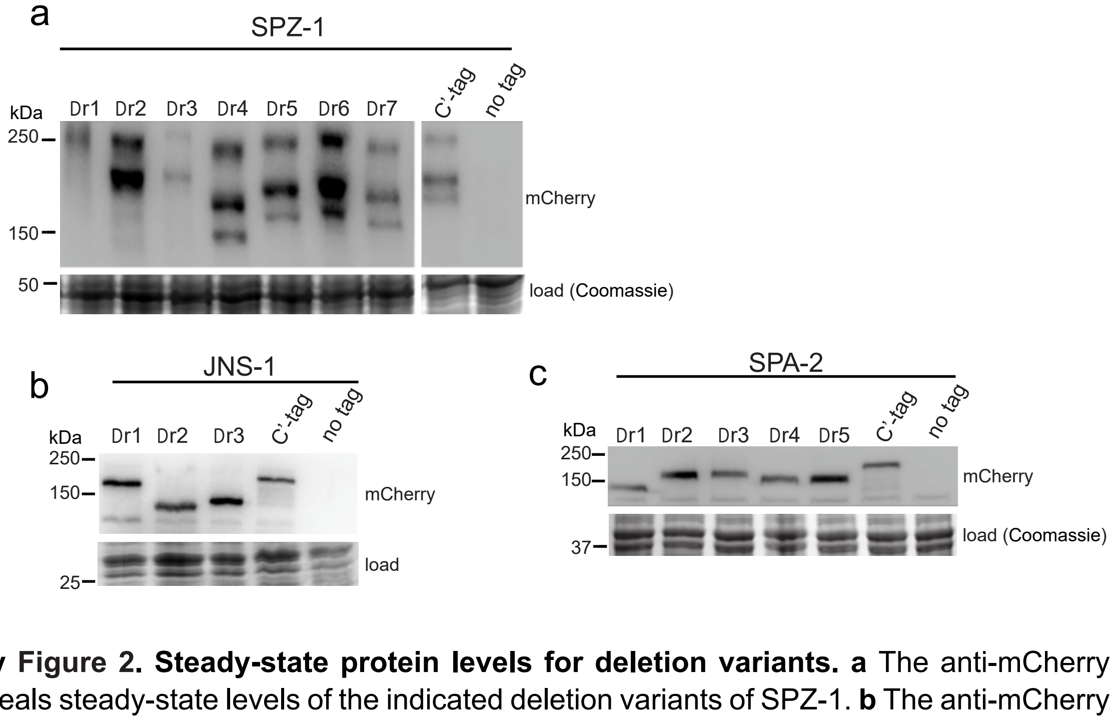

Supplementary Figure 2. Steady-state protein levels for deletion variants. a The anti-mCherry western blot reveals steady-state levels of the indicated deletion variants of SPZ-1. **b** The anti-mCherry western blot reveals steady-state levels of the indicated deletion variants of JNS-1. **c** The anti-mCherry western blot reveals steady-state levels of the indicated deletion variants of SPA-2. A region of a Coomassie stained gel provides a loading control (load). Source data are provided as a Source Data file.

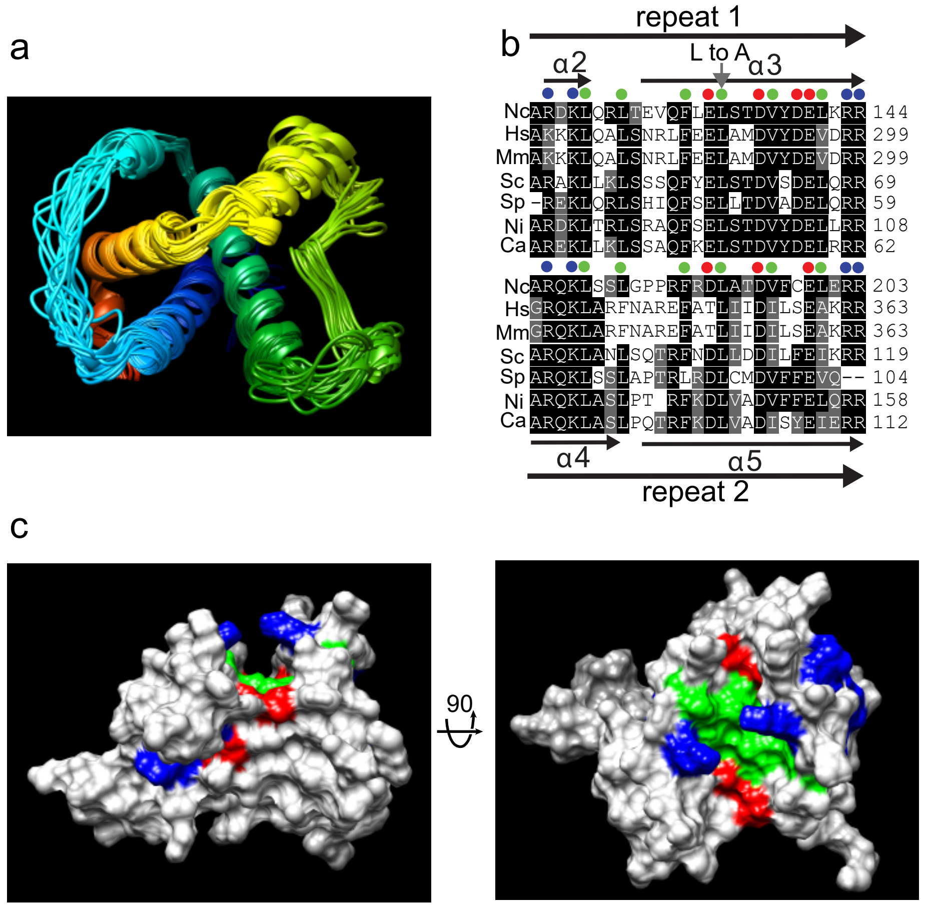

Supplementary Figure 3. Conserved SHD (Spa homology domain) residues map to the SHD surface groove. a Superimposition of 20 SHD low-energy conformations determined by NMR. **b** SHD repeat 1 and 2 are aligned and conserved residues are labelled according to their biophysical character. Basic residues are blue, acidic residues are red, and hydrophobic residues are green. **c** A surface representation of the SHD is shown with conserved residues coloured according to **b**. The perspective of the two panels are the same as shown in Figure 4b and Figure 4c.

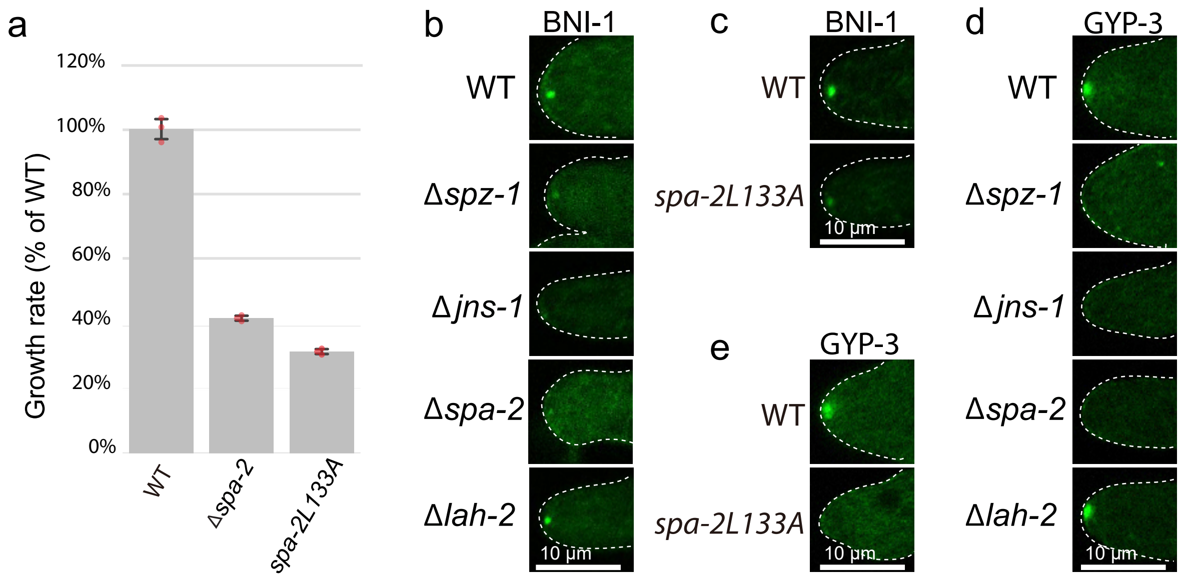

Supplementary Figure 4. Figures related to Figure 4 and 6. a Full loss of function of SPA-2 SHD L133A mutant. For the indicated strains, the average growth rate is shown as mean values ± SD (n = 3 independent measurements). **b** BNI-1 partially depends on SPZ-1, JNS-1 and SPA-2, but not LAH-2. Scale bar = 10 μm. **c** BNI-1 is partially delocalized in the *spa-2L133A* mutant. Scale bar = 10 mm. **d** GYP-3 depends on SPZ-1, JNS-1 and SPA-2, but not LAH-2. Scale bar = 10 mm. **e** GYP-3 is delocalized in the *spa-2L133A* mutant. Scale bar = 10 mm. Source data are provided as a Source Data file.

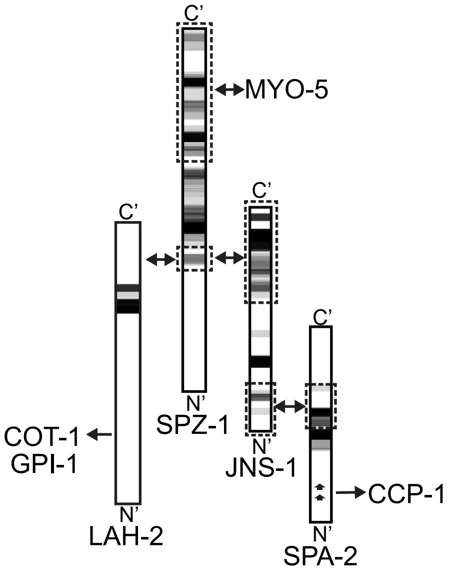

Supplementary Figure 5. Scaffolds are assembled primarily through coiled-coil domains. Coiled coil dimer probability is shown for SPZ-1, JNS-1, SPA-2 and LAH-2. Dashed boxes and arrows indicate regions shown to be required for the indicated interactions.

a

	overall e-value	Domain conditional e-value	
		SHD	CTD
GIT2	7.20E-18	3.50E-14	3.60E-05
GIT1	2.90E-17	1.60E-16	4.30E-03

b

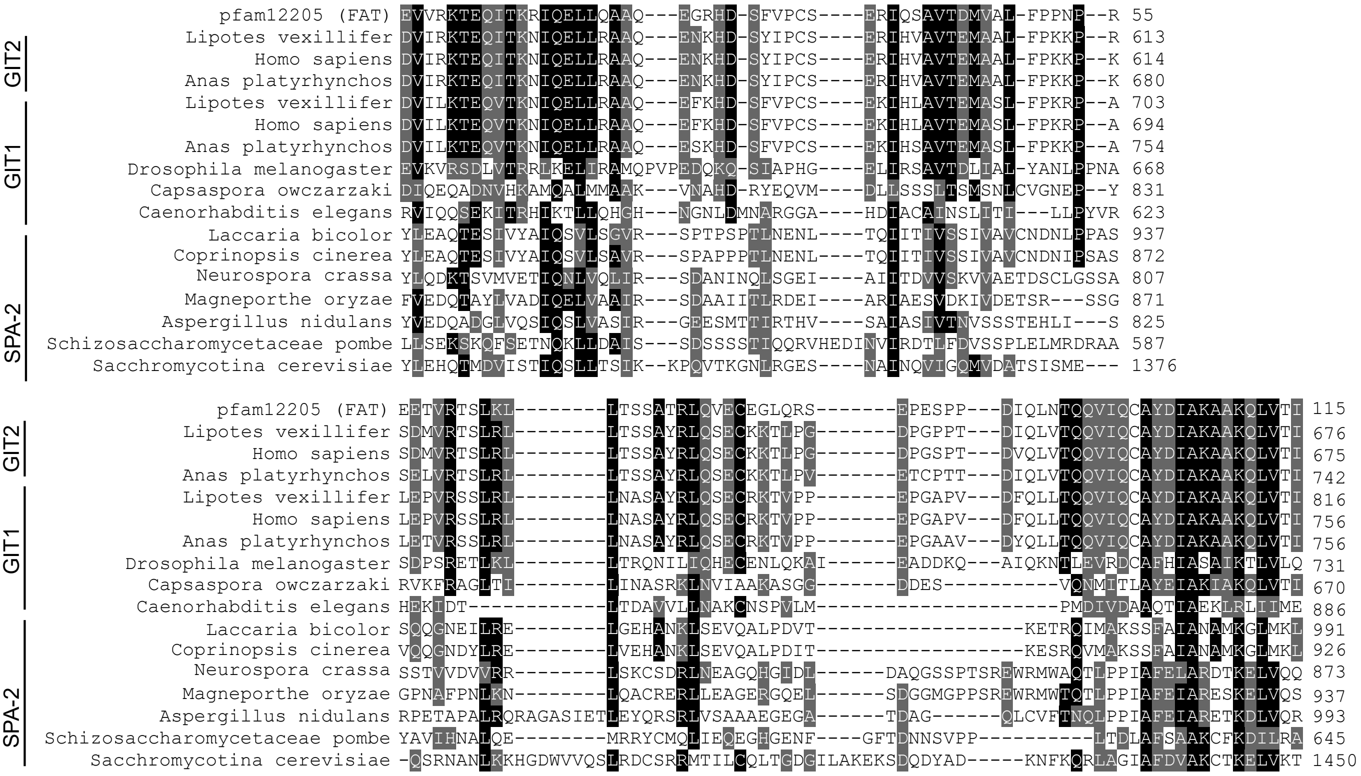

Supplementary Figure 6. Alignment of C-terminal domains of GIT and SPA-2 with the consensus FAT domain (pfam12205). a SPA-2 hmm profiles for the SHD and C-terminal domain (CTD) were constructed from the alignment of Pezizomycotina sequences were used to query the human proteome. The GIT-1 and GIT-2 SHD and FAT domain are identified as significant hits. E-values are shown. **b** Alignment of representative fungal SPA-2 and metazoan C-terminal domains with the pfan12205 FAT domain. Source data are provided as a Source Data file. Identical residues are shaded black, while conserved residues are labeled gray. Numbering corresponds to the full-length proteins.

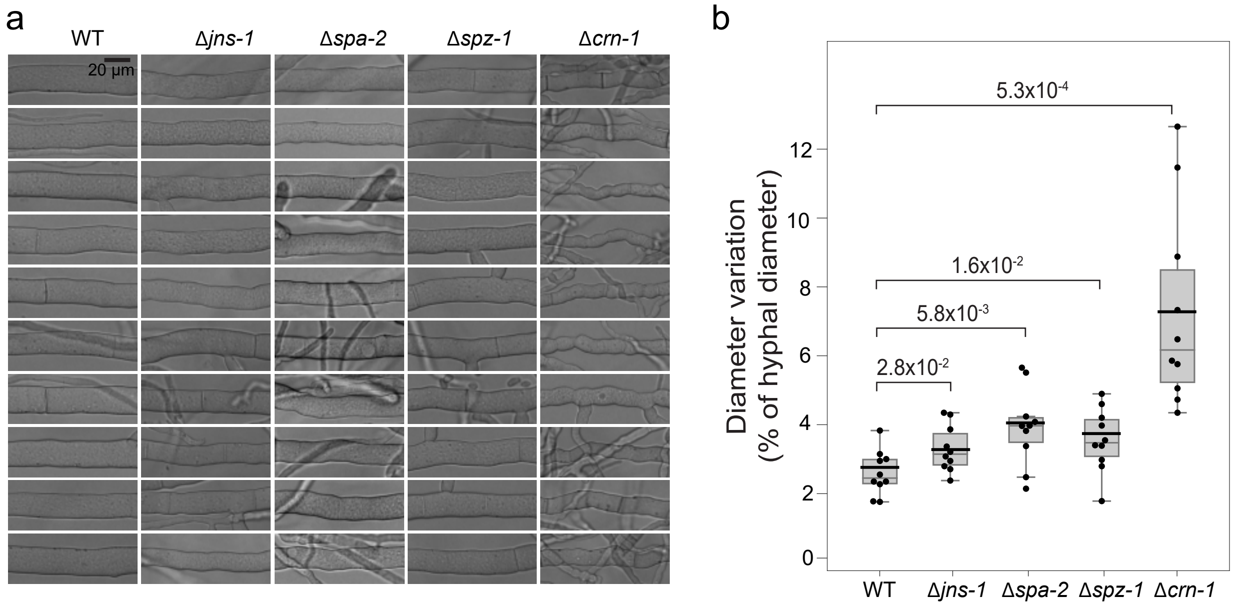

Supplementary Figure 7. *Spz-1*, *jns-1* and *spa-2* deletion mutants do not exhibit major defects in hyphal morphology. a Morphology of representative primary hyphae in the indicated strains. The *crn-1* deletion mutant, which is a known morphological mutant, is used as a positive control. Scale bar = 20 µm. **b** Boxplot showing variation in hyphal diameter quantified from images as shown in **a**. Each data point represents the standard deviation in diameter of one hypha. (median values ± SD, n= 10 independent hyphae).The lower and upper boundaries of each box indicate the first and third quartile, respectively. The whiskers indicate the expected minimum (median - 1.5* interquartile range) and maximum (median + 1.5 * interquartile range). Horizontal black bars indicate the average standard deviation in each group. The p-value of Student's t-test (2 tailed) comparing each mutant to the wild type strain is shown. Source data are provided as a Source Data file.

Supplementary Table 1 (related to Figure 1): Maxquant intensity from co-IP experiments

IP target	**SPZ-1**	**MYO-5**	**JNS-1**	**SPA-2**	**LAH-2**
**SPZ-1-HA**	440740	431110	131500	242960	251350
**MYO-5-HA**	673870	899100	105460	256710	119380
**JNS-1-HA**	37052	781.77	199590	869310	0
**SPA-2-HA**	247570	69865	567220	957620	5198.8
**LAH-2-HA**	11720	0	0	0	20200

Column 1 identifies the IP target. The mass spectrometry maxquant intensity is given for the indicated protiens.

.

Supplementary Table 2 (related to Figure 4):
Restraints used and structural statistics for the final 20 conformers of SHD[a]

Distance restraints	
Intra-residue (i-j = 0)	340
Sequential (|i-j| = 1)	422
Medium range (2 ≤ |i-j| < 4)	193
Long range (|i-j| ≥ 4)	212
Hydrogen bond	110
Total	1277
Dihedral angle restraints (**ϕ, ψ**)	178
Average rmsd to the mean structure (Å)[b]	
Backbone atoms	0.90 ± 0.26
Heavy atoms	1.50 ± 0.25
**ϕ/ψ** space[c]	
Most favored region (%)	94.3
Additionally allowed region (%)	4.9
Generously allowed region (%)	0.7
Disallowed region (%)	0.1
rmsd from covalent geometry	
Bonds (Å)	0.006 ± 0.000
Angles (deg.)	0.794 ± 0.006
Impropers (deg.)	0.405 ± 0.010
rmsd from experimental restraints	
NOEs (Å)	0.047 ± 0.002
Dihedral (deg.)	0.162 ± 0.058
eefx potential energy (kcal)	-1957 ± 21.41

[a] Selected from 100 calculated conformers according to overall energy.
[b] Calculated with MOLMOL (1) using range 6-21, 45-66, 90-101, 104-121, 124-127.
[c] Calculated with PROCHECK-NMR (2).

Supplementary Table 3 (related to Figure 7):

Proteomes used in phylogenetic analyses		
**Species**	**Taxon**	**Reference**
Neurospora crassa	Pezizomycotina	3
Magnaporthe oryzae		4
Fusarium oxysporum		5
Sclerotinia sclerotiorum		3
Aspergillus fumigatus		6
Tuber melanosporum		7
Candida albicans	Saccharomycotina	3
Candida glabrata		8
Saccharomyces cerevisiae		9
Yarrowia lipolytica		8
Schizosaccharomyces pombe	Taphrinomycotina	10
Saitoella complicata		10
Sporobolomyces roseus	Basidiomycota	10
Ustilago maydis		12
Cryptococcus neoformans		13
Coprinopsis cinerea	Agaricomycotina	14
Laccaria bicolor		15
Allomyces macrogynus	Fungi (early diverging)	3
Rhizopus oryzae		16
Homo sapiens	Metazoan	17
Arabidopsis thaliana	Plant	18

Supplementary References

1. Koradi, R., Billeter, M. & Wüthrich, K. MOLMOL: A program for display and analysis of macromolecular structures. *J. Mol. Graph.* **14,** 51–55 (1996).
2. Laskowski, R. A., Rullmann, J. A. C., MacArthur, M. W., Kaptein, R. & Thornton, J. M. AQUA and PROCHECK-NMR: Programs for checking the quality of protein structures solved by NMR. *J. Biomol. NMR* **8,** 477–486 (1996).
3. Broad Institute. Available at: https://www.broadinstitute.org/.
4. Dean, R. A. *et al.* The genome sequence of the rice blast fungus Magnaporthe grisea. **434,** (2005).
5. Ma, L. J., van der Does, H. C., Borkovich, K. a & Al, E. Comparative analysis reveals mobile pathogenicity chromosomes in *Fusarium*. *Nature* **464,** 367–373 (2010).
6. Nierman, W. *et al.* Genomic sequence of the pathogenic and allergenic filamentous fungus Aspergillus fumigatus. *Nature* **438,** 1151–6 (2005).
7. Martin, F. *et al.* Périgord black truffle genome uncovers evolutionary origins and mechanisms of symbiosis. *Nature* **464,** 1033–8 (2010).
8. Dujon, B. *et al.* Genome evolution in yeasts. *Nature* **430,** 35–44 (2004).
9. Goffeau, A. *et al.* Life with 6000 Genes. *Science (80-.).* **274,** 546–567 (1996).
10. Wood, V. *et al.* PomBase: A comprehensive online resource for fission yeast. *Nucleic Acids Res.* **40,** 695–699 (2012).
11. Joint Genome Institute. Available at: http://http//genome.jgi.doe.gov/.
12. Kämper, J. *et al.* Insights from the genome of the biotrophic fungal plant pathogen Ustilago maydis. *Nature* **444,** 97–101 (2006).
13. Loftus, B. J. *et al.* The Genome of the Basidiomycetous Yeast and Human Pathogen Cryptococcus neoformans. *Science (80-.).* **307,** 1321–1324 (2005).
14. Stajich, J. E. *et al.* Insights into evolution of multicellular fungi from the assembled chromosomes of the mushroom Coprinopsis cinerea (Coprinus cinereus). *Pnas* **107,** 11889–11894 (2010).
15. Martin, F. *et al.* The genome of Laccaria bicolor provides insights into mycorrhizal symbiosis. *Nature* **452,** 88–92 (2008).
16. Ma, L. J. *et al.* Genomic analysis of the basal lineage fungus Rhizopus oryzae reveals a whole-genome duplication. *PLoS Genet.* **5,** (2009).
17. Lander, E. S. *et al.* Initial sequencing and analysis of the human genome. *Nature* **409,** 860–921 (2001).
18. The Arabidopsis Genome Initiative. Analysis of the genome sequence of the flowering plant Arabidopsis thaliana. *Nature* **408,** 796–815 (2000).
