# Supplementary material for: Spitzenkörper assembly mechanisms reveal conserved features of fungal and metazoan polarity scaffolds
Source: Nat Commun. 2020 Jun 5;11:2830. doi: 10.1038/s41467-020-16712-9 (PMC7275032; doi:10.1038/s41467-020-16712-9)
Supplement: Supplementary file 3 — Description of Additional Supplementary Files [file 41467_2020_16712_MOESM3_ESM.pdf]

Description of Additional Supplementary Files

File Name: Supplementary Data 1
Description: *Neurospora crassa* strains used in this study. The genotype and origin of the strains used in this paper is given according to the figure they are associated with. In the case of deletion variants produced by marker fusion tagging (MFT), the numbers following the deletion symbol (Δ) refer to the range of amino-acid replaced by the MFT marker.
