# Supplementary material for: Spitzenkörper assembly mechanisms reveal conserved features of fungal and metazoan polarity scaffolds
Source: Nat Commun. 2020 Jun 5;11:2830. doi: 10.1038/s41467-020-16712-9 (PMC7275032; doi:10.1038/s41467-020-16712-9)
Supplement: Supplementary file 5 — Reporting Summary [file 41467_2020_16712_MOESM5_ESM.pdf]

nature research

Corresponding author(s): Gregory JEDD
Last updated by author(s): May 7, 2020

Reporting Summary

Nature Research wishes to improve the reproducibility of the work that we publish. This form provides structure for consistency and transparency in reporting. For further information on Nature Research policies, see Authors & Referees and the Editorial Policy Checklist.

Statistics

For all statistical analyses, confirm that the following items are present in the figure legend, table legend, main text, or Methods section.

n/a	Confirmed	
☐	☒	The exact sample size (n) for each experimental group/condition, given as a discrete number and unit of measurement
☐	☒	A statement on whether measurements were taken from distinct samples or whether the same sample was measured repeatedly
☐	☒	The statistical test(s) used AND whether they are one- or two-sided<br>*Only common tests should be described solely by name; describe more complex techniques in the Methods section.*
☐	☒	A description of all covariates tested
☐	☒	A description of any assumptions or corrections, such as tests of normality and adjustment for multiple comparisons
☐	☒	A full description of the statistical parameters including central tendency (e.g. means) or other basic estimates (e.g. regression coefficient) AND variation (e.g. standard deviation) or associated estimates of uncertainty (e.g. confidence intervals)
☐	☒	For null hypothesis testing, the test statistic (e.g. F, t, r) with confidence intervals, effect sizes, degrees of freedom and P value noted<br>*Give P values as exact values whenever suitable.*
☒	☐	For Bayesian analysis, information on the choice of priors and Markov chain Monte Carlo settings
☒	☐	For hierarchical and complex designs, identification of the appropriate level for tests and full reporting of outcomes
☒	☐	Estimates of effect sizes (e.g. Cohen's d, Pearson's r), indicating how they were calculated

Our web collection on statistics for biologists contains articles on many of the points above.

Software and code

Policy information about availability of computer code

Data collection	1. Confocal images were acquired using Leica Appkication Suite X (LAS X 3.5.5.19976) software.<br>2. Gels and blots were imaged using ChemiDoc™ Touch (Bio-RAD) software.<br>3. NMR spectra were recorded with a Bruker Avance 800 spectrometer using TOPSPIN software (www.bruker.de)
Data analysis	1. Maxquant (https://www.maxquant.org) was used for analysis of co-IP data.<br>2. Images were analyzed using ImageJ (released 2017 May 30, https://imagej.net/Fiji/Downloads).<br>3. Images were cropped and brightness was adjusted using the curves function in Adobe Photoshop CS6.<br>4. Figures were generated using Adobe Illustrator CS6.<br>5. Protein sequences were aligned with MAFFT v6.240.<br>6. Sequence alignments were trimmed TrimAl v1.1.<br>7. Maximum likelihood phylogenies were inferred using RAxML version 8.1.15.<br>8. NMR spectra were processed using NMRPipe v10.8.<br>9. NMR spectra were analyzed using Sparky v3.108.

For manuscripts utilizing custom algorithms or software that are central to the research but not yet described in published literature, software must be made available to editors/reviewers. We strongly encourage code deposition in a community repository (e.g. GitHub). See the Nature Research guidelines for submitting code & software for further information.

Data

Policy information about availability of data

All manuscripts must include a data availability statement. This statement should provide the following information, where applicable:

- Accession codes, unique identifiers, or web links for publicly available datasets
- A list of figures that have associated raw data
- A description of any restrictions on data availability

A reporting summary for this article is available as a Supplementary Information file. The source data underlying Figs. 1c-f; 2a-c; 3b, e, f, h, i; 5b-d, f; 6a-c and

Supplementary Figs. 1a, b; 2a-c and 4a-e are provided in the Source data file. The N. crassa SPA-2 Spa Homology Domain (SHD) NMR structure is deposited with the Protein Data Bank under accession number PBD 6LAG [DOI: 10.2210/pdb6LAG/pdb] and the assigned chemical shifts are deposited with the Biological Magnetic Resonance Bank under accession number BMRB ID 36299 [10.13018/BMR36299]. Data supporting the findings of this manuscript are available from the corresponding author upon reasonable request.

Field-specific reporting

Please select the one below that is the best fit for your research. If you are not sure, read the appropriate sections before making your selection.

☒ Life sciences ☐ Behavioural & social sciences ☐ Ecological, evolutionary & environmental sciences

For a reference copy of the document with all sections, see nature.com/documents/nr-reporting-summary-flat.pdf

Life sciences study design

All studies must disclose on these points even when the disclosure is negative.

Sample size	For growth rate comparison, 3 measurements were taken each strain. For image analyses, at least 5 independent images were taken for each strain.
Data exclusions	No data were excluded.
Replication	Experiments were replicated at least 3 times.
Randomization	None of the experimental designs required randomization.
Blinding	None of the experimental designs required blinding.

Reporting for specific materials, systems and methods

We require information from authors about some types of materials, experimental systems and methods used in many studies. Here, indicate whether each material, system or method listed is relevant to your study. If you are not sure if a list item applies to your research, read the appropriate section before selecting a response.

Materials & experimental systems

n/a	Involved in the study	
☐	☒	Antibodies
☒	☐	Eukaryotic cell lines
☒	☐	Palaeontology
☒	☐	Animals and other organisms
☒	☐	Human research participants
☒	☐	Clinical data

Methods

n/a	Involved in the study	
☒	☐	ChIP-seq
☒	☐	Flow cytometry
☒	☐	MRI-based neuroimaging

Antibodies

Antibodies used	anti-HA-HRP antibody (INVITROGEN, 26183-HRP)<br>anti-HA epitope 3FA antibody (Roche, 11867423001)<br>Mouse monoclonal antibody to RFP(Chromotek, 6G6)<br>Goat anti mouse HRP (GE, NA931; RRID: AB-72210)
Validation	Information of anti-HA-HRP antibody validation is available at the product website: https://www.thermofisher.com/antibody/product/HA-Tag-Antibody-clone-2-2-2-14-Monoclonal/26183-HRP<br>Information of anti-HA epitope 3FA antibody validation is available at the product website: https://www.sigmaaldrich.com/catalog/product/roche/roahaha?lang=en&region=SG<br>Mouse monoclonal antibody to RFP(Chromotek, 6G6) antibody validation is available at the product website: https://www.chromotek.com/products/detail/product-detail/rfp-antibody-6g6/<br>Goat anti mouse HRP (GE, NA931; RRID: AB-72210) antibody validation is available at the product website: https://antibodyregistry.org/search.php?q=AB_772210
